# Phase 1b clinical trial of ado-trastuzumab emtansine and ribociclib for HER2-positive metastatic breast cancer

**DOI:** 10.1038/s41523-021-00311-y

**Published:** 2021-08-04

**Authors:** Laura M. Spring, Shealagh L. Clark, Tianyu Li, Shom Goel, Nabihah Tayob, Elene Viscosi, Elizabeth Abraham, Dejan Juric, Steven J. Isakoff, Erica Mayer, Beverly Moy, Jeffrey G. Supko, Sara M. Tolaney, Aditya Bardia

**Affiliations:** 1 Massachusetts General Hospital Cancer Center, Harvard Medical School, Boston, MA USA; 2 Dana-Farber Cancer Institute, Harvard Medical School, Boston, MA USA

**Keywords:** Breast cancer, Targeted therapies

## Abstract

Patients with HER2+ metastatic breast cancer are often treated with a multitude of therapies in the metastatic setting, and additional strategies to prolong responses to anti-HER2 therapies are needed. Preclinical evidence suggests synergy between cyclin-dependent kinase 4 and 6 (CDK 4/6) inhibitors and anti-HER2 therapies. We conducted a phase 1b study of ribociclib and ado-trastuzumab emtansine (T-DM1) in patients with advanced/metastatic HER2-positive breast cancer previously treated with trastuzumab and a taxane in any setting, with four or fewer prior lines of therapy in the metastatic setting. A standard 3 + 3 dose-escalation design was used to evaluate various doses of ribociclib in combination with T-DM1, starting at 300 mg. The primary objective was to determine the maximum tolerated dose and/or recommended phase 2 dose (RP2D) of ribociclib in combination with T-DM1. A total of 12 patients were enrolled. During dose-escalation, patients received doses of ribociclib of 300 mg (*n* = 3), 400 mg (*n* = 3), 500 mg (*n* = 3), and 600 mg (*n* = 3). No dose-limiting toxicities were observed. The majority of toxicities were Grade 1 and 2, and the most common Grade 3 toxicities were neutropenia (33%), leukopenia (33%), and anemia (25%). After a median follow-up of 12.4 months, the median PFS was 10.4 months (95% confidence interval, 2.7–19.3). Based on the pharmacokinetic analysis, adverse events, and dose reductions, 400 mg was determined to be the RP2D for ribociclib given on days 8–21 of a 21-day cycle with T-DM1.

## Introduction

The presence of human epidermal growth factor receptor 2 (HER2), also known as ERBB2, amplification confers an aggressive breast cancer phenotype. However, anti-HER2-directed therapies have changed the landscape and prognosis of HER2-positive (HER2+) breast cancer. Despite the development of multiple effective therapies for HER2-positive breast cancer, nearly all patients with advanced/metastatic HER2-positive disease will eventually experience disease progression and subsequent death^1^. Approved anti-HER2 therapies in the metastatic setting include HER2 monoclonal antibodies (trastuzumab, pertuzumab), tyrosine kinase inhibitors (lapatinib, neratinib, tucatinib), and antibody-drug conjugates (ado-trastuzumab emtansine/T-DM1, trastuzumab deruxtecan)^2–10^. Patients with HER2+ metastatic breast cancer (MBC) are living longer, often with good performance status, allowing additional therapies to be considered.

Combinations involving targets downstream of the HER2 pathway, particularly cyclin D and cyclin-dependent kinases (CDK) 4/6, could potentially enhance therapeutic efficacy in HER2+ MBC. While CDK 4/6 inhibitors (palbociclib, ribociclib, abemaciclib) have been FDA approved for the treatment of hormone receptor-positive (HR+) MBC^11^, the activity has been attributed in part to the proven pathogenesis of these tumors in promoting cyclin D1 expression and CDK 4/6 activity^12,13^. This activity also occurs downstream of HER2, thus making it an appealing partner with anti-HER2 therapy^12^. Indeed, the malignant transformation of mammary epithelial cells by HER2 is dependent on cyclin D1, and genetic ablation of cyclin D1 in mice makes them resistant to tumors induced by the neu oncogene^12^. Besides the role in HR+ MBC, pre-clinical data suggests that the cyclin D1-CDK4/6-Rb axis may be an attractive therapeutic target in HER2+ breast cancer as well. Using transgenic mouse models, cell line-based functional studies, and clinical specimens, Goel and colleagues demonstrated that heightened activity of the CDK4/6 pathway mediated resistance to HER2-targeted therapies which could be overcome by the use of a CDK 4/6 inhibitor^14^. It has also been observed that tumor cells that survive HER2-blockade retain a high expression of cyclin D1 and, when targeted with CDK 4/6 inhibitors, become re-sensitized to anti-HER2 therapy by not only reducing the retinoblastoma phosphorylation, but also by suppressing mTORC1/S6K/S6RP activity and increasing tumor cell dependence on epidermal growth factor receptor family kinases^14–16^.

Pre-clinical work has also demonstrated CDK 4/6 inhibition provided a complementary mechanism of action to T-DM1, and efficiently suppressed the proliferation of residual HER2-positive tumor cell populations that survived T-DM1^15^. Theoretically, the inhibition of cellular division by CDK 4/6 inhibitors could potentially reduce the efficacy of chemotherapy by preventing cells from entering S or M phase^17^. The half-life of DM1 is approximately 4 days^18^, suggesting the optimal strategy for combining T-DM1 and CDK 4/6 inhibition would be to avoid a concurrent dosing strategy. Such an approach may also reduce overlapping toxicities, such as thrombocytopenia. In addition, pre-clinical work suggests CDK 4/6 inhibitors impair recovery from chemotherapy-induced chromosomal damage, supporting sequential use^19^.

Initial clinical studies have reported results of CDK 4/6 inhibition combined with select anti-HER2 therapies. The monarcHER trial, a randomized open-label phase 2 study of the CDK 4/6 inhibitor abemaciclib plus trastuzumab with or without fulvestrant versus trastuzumab plus standard-of-care chemotherapy in women with HR+/HER2+ MBC demonstrated the combination with abemaciclib significantly improved progression-free survival compared to the chemotherapy arm while exhibiting a tolerable safety profile^20^. The monarcHER study was notably limited to patients with “triple positive” (HR+/HER2+) disease. In a phase 1b/2 trial of ribociclib (continuous dosing, 400 mg per day) plus trastuzumab in heavily pretreated patients with HER2+ MBC, limited clinical activity was observed with a median PFS of 1.33 months^21^. The recently reported phase II SOLTI-1303 PATRICIA trial further supports that the activity of CDK 4/6 inhibitors in HER2+ MBC is greater in patients who are also HR+^22^.

Given the pre-clinical observations and strong rationale behind the dual vertical blockade, we conducted a phase 1b study of the CDK 4/6 inhibitor ribociclib and T-DM1 in patients with HER2+ MBC, specifically with a staggered dosing schedule. Here we report the results of the clinical trial, including the safety and efficacy of the combination therapy.

## Results

### Patient characteristics

Between March 9, 2016, and May 25, 2019, a total of 12 patients were enrolled in this cohort (cohort A) of the study. Accrual to the study was closed in 2019 due to slow accrual and sponsor decisions based on prioritization of internal resources. Baseline patient and disease characteristics are listed in Table 1. Overall median age at inclusion was 52 years old (range, 37–71 years), and most patients (67%) had ER and/or PR-positive disease. The median number of prior lines of systemic therapy for metastatic disease was 2 (range, 0–3) with nine (75%) and seven patients (58.3%) having had prior exposure to pertuzumab and T-DM1, respectively.

**Table 1:** Patient characteristics.

Characteristics	Patients, no. (%) *N* = 12
Age (median, range), years	52 (37–71)
Race/ethnicity	
White	7 (58.3%)
ECOG performance status	
0	8 (66.7%)
1	4 (33.3%)
ER and/or PR positive	8 (66.7%)
Number of metastatic sites (median, range)	2 (1–5)
Previous pertuzumab in any setting	9 (75%)
Previous T-DM1 in any setting	7 (58.3%)
Number of prior lines of therapy for metastatic disease (median, range)	2 (0–3)

### Dose escalation and adverse events (AEs)

During dose escalation, patients received doses of ribociclib of 300 mg (*n* = 3), 400 mg (*n* = 3), 500 mg (*n* = 3), and 600 mg (*n* = 3). No DLTs were observed. Of the twelve patients, seven (58.3%) required a dose reduction (dose level-1) of ribociclib while on study due to thrombocytopenia or neutropenia. Three of these patients later required another dose reduction (dose level-2) due to recurring toxicity, most commonly grade 3 neutropenia.

The observed toxicities or AEs are listed in Table 2, the majority of which were grade 1 or 2. AEs by dose level are displayed in Supplementary Table 1a–d. Observed Grade 3 toxicities included neutropenia (33%), leukopenia (33%), anemia (25%), lymphopenia (16.67%), thrombocytopenia (16.67%), febrile neutropenia (8.33%), elevated transaminases (8.33%), hypophosphatemia (8.33%), and urinary tract infection (8.33%). It should be noted that the dosage schedule of ribociclib was changed from Days 5–18 to Days 8–21 of a 21-day cycle due to thrombocytopenia in the setting of concurrent T-DM1 therapy. During cycle 1, prior to the change in dosing schedule, two patients at the 300 mg dose level had ribociclib temporarily held due to Grade 3 thrombocytopenia attributed to T-DM1. One patient (8.33%) experienced grade 4 neutropenia. No other grade 4 toxicities were reported. The greatest frequency of grade 3 AEs occurred at the highest dose level (600 mg) with a total of 11, while four grade 3 AEs occurred at 500 mg and only one grade 3 AE at a dose level of 400 mg. One patient (8.33%) demonstrated a significant increase in QTcF prolongation (>480 ms; Grade 2 AE) without complications at the 600 mg dose level and no patients experienced a significant change in LVEF (defined as LVEF <50%).

**Table 2:** Treatment-related adverse events (AE).

Adverse event (AE) type	AE of any grade	Grade 1 AE	Grade 2 AE	Grade 3 AE	Grade 4 AE
Platelet count decreased	11(91.67%)	5(41.67%)	4(33.33%)	2(16.67%)	0(0%)
Anemia	10(83.33%)	4(33.33%)	3(25%)	3(25%)	0(0%)
Neutrophil count decreased	9(75%)	0(0%)	4(33.33%)	4(33.33%)	1(8.33%)
White blood cell decreased	8(66.67%)	2(16.67%)	2(16.67%)	4(33.33%)	0(0%)
Fatigue	7(58.33%)	4(33.33%)	3(25%)	0(0%)	0(0%)
Aspartate aminotransferase increased	6(50%)	3(25%)	2(16.67%)	1(8.33%)	0(0%)
Alanine aminotransferase increased	5(41.67%)	3(25%)	1(8.33%)	1(8.33%)	0(0%)
Electrocardiogram QT corrected interval prolonged	5(41.67%)	4(33.33%)	1(8.33%)	0(0%)	0(0%)
Lymphocyte count decreased	5(41.67%)	2(16.67%)	1(8.33%)	2(16.67%)	0(0%)
Nausea	5(41.67%)	4(33.33%)	1(8.33%)	0(0%)	0(0%)
Blood bilirubin increased	3(25%)	2(16.67%)	1(8.33%)	0(0%)	0(0%)
Cough	3(25%)	3(25%)	0(0%)	0(0%)	0(0%)
Diarrhea	3(25%)	2(16.67%)	1(8.33%)	0(0%)	0(0%)
Epistaxis	3(25%)	3(25%)	0(0%)	0(0%)	0(0%)
Mucositis oral	3(25%)	3(25%)	0(0%)	0(0%)	0(0%)
Alkaline phosphatase increased	2(16.67%)	2(16.67%)	0(0%)	0(0%)	0(0%)
Bruising	2(16.67%)	2(16.67%)	0(0%)	0(0%)	0(0%)
Creatinine increased	2(16.67%)	0(0%)	2(16.67%)	0(0%)	0(0%)
Dry mouth	2(16.67%)	1(8.33%)	1(8.33%)	0(0%)	0(0%)
Fever	2(16.67%)	0(0%)	2(16.67%)	0(0%)	0(0%)
Hypophosphatemia	2(16.67%)	0(0%)	1(8.33%)	1(8.33%)	0(0%)
Rash maculo-papular	2(16.67%)	2(16.67%)	0(0%)	0(0%)	0(0%)
Vomiting	2(16.67%)	1(8.33%)	1(8.33%)	0(0%)	0(0%)
Weight loss	2(16.67%)	2(16.67%)	0(0%)	0(0%)	0(0%)
Activated partial thromboplastin time prolonged	1(8.33%)	0(0%)	1(8.33%)	0(0%)	0(0%)
Alopecia	1(8.33%)	1(8.33%)	0(0%)	0(0%)	0(0%)
Arthritis	1(8.33%)	1(8.33%)	0(0%)	0(0%)	0(0%)
Bloating	1(8.33%)	1(8.33%)	0(0%)	0(0%)	0(0%)
Constipation	1(8.33%)	1(8.33%)	0(0%)	0(0%)	0(0%)
Cystitis noninfective	1(8.33%)	1(8.33%)	0(0%)	0(0%)	0(0%)
Dry eye	1(8.33%)	1(8.33%)	0(0%)	0(0%)	0(0%)
Febrile neutropenia	1(8.33%)	0(0%)	0(0%)	1(8.33%)	0(0%)
Gastroesophageal reflux disease	1(8.33%)	0(0%)	1(8.33%)	0(0%)	0(0%)
Gastrointestinal disorders—other, specify	1(8.33%)	0(0%)	1(8.33%)	0(0%)	0(0%)
Headache	1(8.33%)	1(8.33%)	0(0%)	0(0%)	0(0%)
Hyperhidrosis	1(8.33%)	1(8.33%)	0(0%)	0(0%)	0(0%)
Hypokalemia	1(8.33%)	1(8.33%)	0(0%)	0(0%)	0(0%)
Infusion related reaction	1(8.33%)	1(8.33%)	0(0%)	0(0%)	0(0%)
Lung infection	1(8.33%)	0(0%)	1(8.33%)	0(0%)	0(0%)
Myalgia	1(8.33%)	1(8.33%)	0(0%)	0(0%)	0(0%)
Oral hemorrhage	1(8.33%)	1(8.33%)	0(0%)	0(0%)	0(0%)
Pain	1(8.33%)	1(8.33%)	0(0%)	0(0%)	0(0%)
Peripheral motor neuropathy	1(8.33%)	1(8.33%)	0(0%)	0(0%)	0(0%)
Renal and urinary disorders—other, specify	1(8.33%)	0(0%)	0(0%)	1(8.33%)	0(0%)
Salivary duct inflammation	1(8.33%)	1(8.33%)	0(0%)	0(0%)	0(0%)
Thrombotic thrombocytopenic purpura	1(8.33%)	0(0%)	1(8.33%)	0(0%)	0(0%)
Upper respiratory infection	1(8.33%)	0(0%)	1(8.33%)	0(0%)	0(0%)
Urinary tract infection	1(8.33%)	0(0%)	0(0%)	1(8.33%)	0(0%)

### Efficacy

**Fig. 1:**
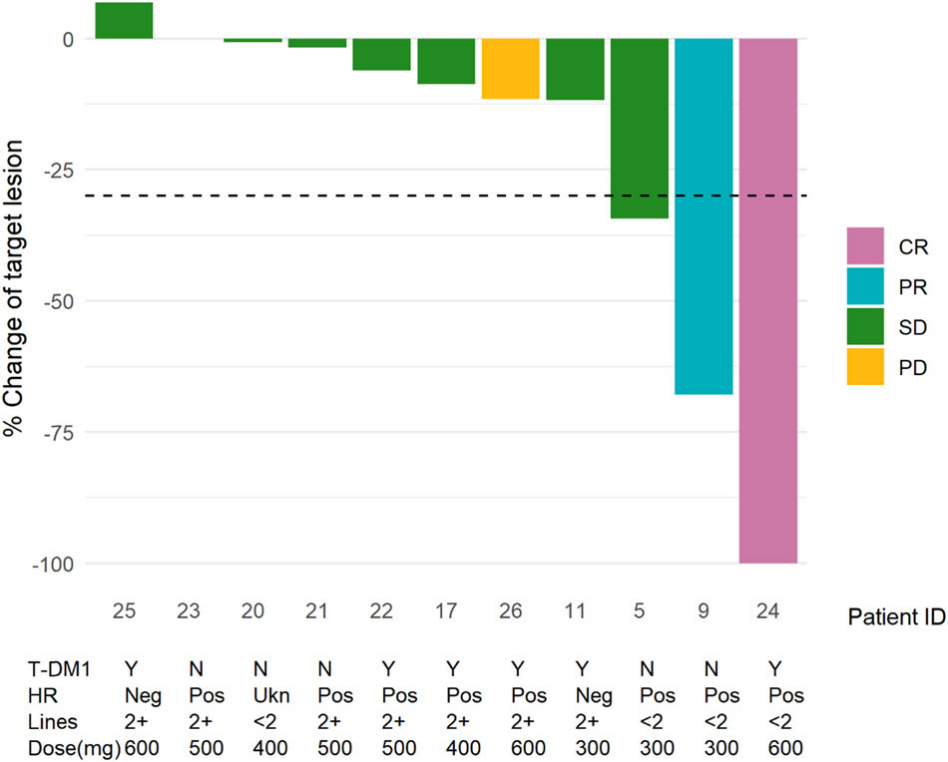
Waterfall plot demonstrating objective response rates for study participants. The median follow-up time was 12.4 months (95% CI: 2.7–15.4). One patient without a target lesion was excluded from this figure. Prior T-DM1 use (Y yes, N no), hormone receptor (HR) status, number of lines of prior therapy, and assigned dose of ribociclib are notated below the *X*-axis. PD progressive disease, CR complete response, PR partial response, SD stable disease, Neg negative, Pos positive, Ukn unknown.

In terms of efficacy, one patient (8.3%) experienced a complete response, one patient (8.3%) experienced a partial response, and nine patients (75%) experienced stable disease (Fig. 1). The ORR was therefore 16.7% (90% CI 3-44%). Of the nine patients who experienced stable disease, two (16.7%) experienced stable disease for less than 12 weeks and seven (58.3%) experienced stable disease for greater than or equal to 24 weeks of therapy. One patient (8.3%) had progressive disease per RECIST 1.1. Supplementary Fig. 1 demonstrates each subject’s response to treatment in a swimmer plot.

**Fig. 2:**
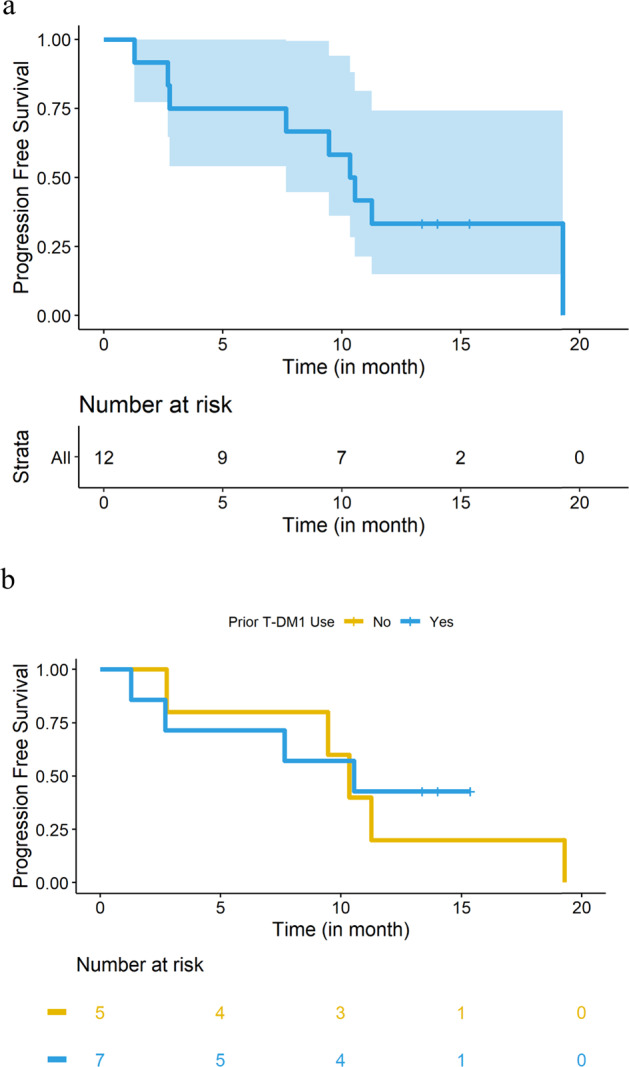
Progression-free survival (PFS) results. **a** Median PFS summarized in a Kaplan–Meier plot. Median PFS was 10.4 months (95% CI [2.7–19.3]). **b** Median PFS summarized in a Kaplan–Meier plot, stratified by prior T-DM1 use. The median PFS for patients with and without prior T-DM1 use was 10.5 months (95% CI [1.3-not reached]) and 10.3 months (95% CI [2.8–19.3]), respectively.

After a median follow-up of 12.4 months, the median PFS was 10.4 months (95% confidence interval, 2.7–19.3) (Fig. 2a). The median PFS for patients with and without prior T-DM1 use was 10.5 months (95% CI [1.3-not reached]) and 10.3 months (95% CI [2.8–19.3]), respectively (Fig. 2b). The median PFS for patients with and without HR positivity was 10.8 months (95% CI [1.3–19.3]) for HR+, and 10.5 months (95% CI [2.7-not reached]) for HR-, respectively (Supplementary Figure 2).

### Pharmacokinetic data

Geometric mean (CV%) values of the ribociclib pharmacokinetic parameters are presented in Supplementary Table 2. Pharmacokinetic data for the first dose of the drug was obtained for all three patients in each of the four dose levels evaluated. The *C*_max_ was significantly correlated with the dose (*r* = 0.606, *P* = 0.037) although AUC_24_ did not exhibit a significant trend toward increasing values as the dose was escalated from 300 to 600 mg (*r* = 0.486, *P* = 0.11). Data on the steady-state pharmacokinetics of ribociclib was available for only 7 of the patients. The steady-state *C*_max_ (*r* = 0.728, *P* = 0.064) and AUC_24_ (*r* = 0.735, *P* = 0.060) were both moderately correlated with the dose but neither association was statistically significant, most likely because of the low number of patients. The overall geometric mean (CV%) apparent oral clearance of ribociclib in these patients, determined from the AUC24 at steady state, was 42.1 L/h (28.1%). Based on the pharmacokinetic data, adverse events, and dose reductions, 400 mg was determined to be the RP2D with T-DM1.

### Circulating tumor DNA assessment

**Fig. 3:**
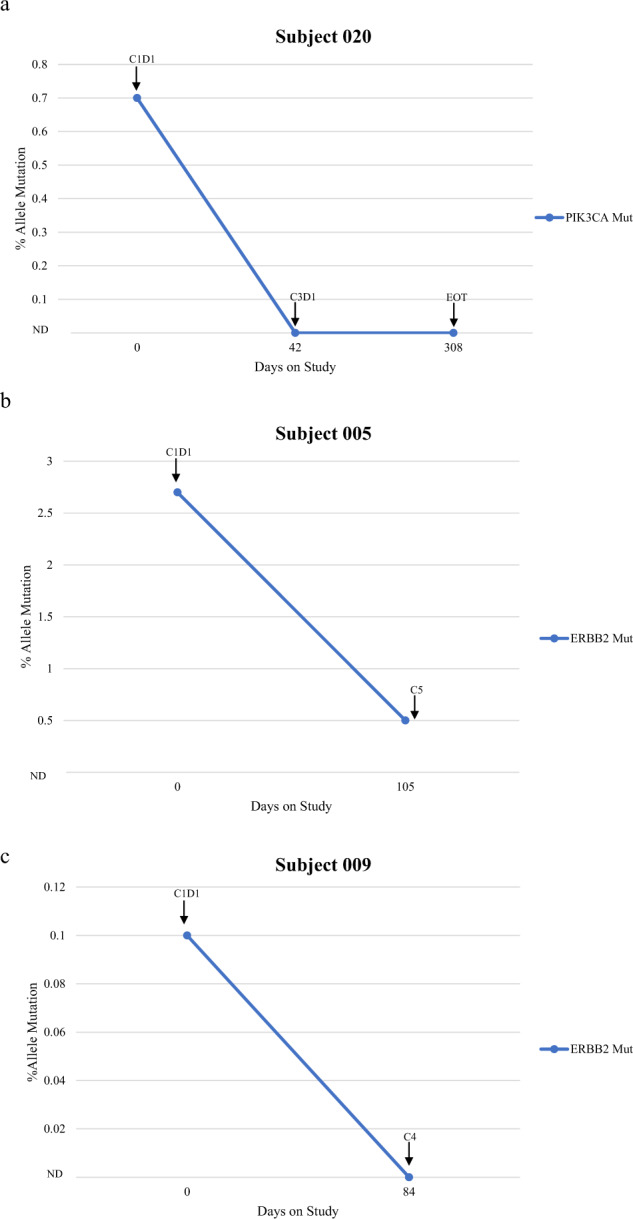
Select circulating tumor DNA (ctDNA) results. **a** Subject 020 ctDNA results. C cycle, D day, ND not detected, EOT end of treatment, Mut mutation. **b** Subject 005 ctDNA results. C cycle, D day, ND, not detected, Mut mutation. **c** Subject 009 ctDNA results. C cycle, D day, ND not detected, Mut mutation.

ctDNA samples were collected prior to initiation of therapy in nine patients (75%) as shown in Supplementary Fig. 3. TP53 alterations were the most common baseline molecular alteration observed (*n* = 5). The median number of alterations at baseline was 2 (range 1–9). Four patients had ERBB2 alterations (mutations and/or amplification) at baseline. Serial ctDNA monitoring can provide evidence of pharmacodynamic inhibition as well as evidence of molecular progression before radiological progression^23^. Patient 020 had a high level (+++) ERBB2 amplification that became undetectable by C2 and remained undetectable post-treatment, and also had resolution of a PIK3CA mutation and FGFR1 amplification, highlighting evidence of molecular response with therapy (Fig. 3a). Similarly, patient 005 had a baseline ERBB2 alteration with an allelic fraction of 2.7% at baseline, which was observed to decrease to 0.5% on treatment at cycle 5 (Fig. 3b), and patient 009 had a baseline ERBB2 alteration with an allelic fraction of 0.1% and low level (+) ERBB2 amplification at baseline, both of which was undetectable on treatment when assessed at cycle 4 (Fig. 3c).

## Discussion

To our knowledge, this is the first study to investigate the use of ribociclib in combination with T-DM1 in locally advanced/metastatic HER2+ breast cancer. The results from this study support the pre-clinical data in suggesting the combination of a CDK 4/6 inhibitor and T-DM1 is active with a median PFS of 10.4 months. Ribociclib in combination with T-DM1 was generally well-tolerated with reversible, expected hematologic toxicities and showed promising clinical activity in patients who had progressive disease on prior anti-HER2 therapy. While the MTD of ribociclib in combination with T-DM1 was 600 mg, based on PK analysis and dose reductions, 400 mg of ribociclib was determined to be the RP2D with T-DM1. The major overlapping toxicity between ribociclib and T-DM1 is thrombocytopenia and transaminitis^24,25^.

The study had a few limitations. This study had a limited sample size (*N* = 12), and further exploration is warranted. In addition, while the median PFS was 10.4 months, a randomized study would be needed to better delineate the contribution of ribociclib given the known efficacy of T-DM1 in this population. In the KATE2 study comparing T-DM1 plus atezolizumab versus T-DM1 plus placebo in patients with HER2+ advanced breast cancer previously treated with a taxane and trastuzumab (and 48% also received pertuzumab), the median PFS in the control group was 6.8 months^26^. Our study population was heterogeneous, with a subset of patients receiving prior T-DM1. Accordingly, we conducted efficacy analysis stratified by prior T-DM1. Finally, the small sample size limits the interpretation of the ctDNA results, where technical and biological factors (such as shedding dynamics) could influence results. Confirmation in a larger study is warranted.

Systemic exposure to ribociclib tended to be lower in the patients evaluated in this study than previously observed when given alone to patients with solid tumors in phase I clinical trial^27,28^. When given as a single agent, the geometric mean (CV%) apparent oral clearance was found to be 35.3 L/h (59.2%) in four patients treated with ribociclib 400 mg QD and 25.5 L/h (65.7%) in 53 patients treated with the approved dose of 600 mg QD. In comparison, the overall geometric mean apparent oral clearance of ribociclib was 19–65% greater (42.1 L/h (28.1%)) in the seven patients for whom steady-state pharmacokinetic data were available in the present clinical trial. Hepatic metabolism, predominantly mediated by CYP3A4, is an important route of elimination for ribociclib^29^. It appears hepatic first-pass metabolism by CYP3A4 can limit the oral bioavailability of ribociclib and contribute to high variation in systemic exposure to the drug when given orally^30^. Regardless, it is unlikely that the administration of a single dose of trastuzumab emtansine on day 1 of cycle 1 could affect the steady-state pharmacokinetics of ribociclib as determined from samples for a dose given approximately two weeks later. A nonclinical study demonstrated that radiolabeled emtansine is completely cleared from rats within 5 days after administering a single dose of the conjugate with trastuzumab^31^. In addition, it has been shown that the pharmacokinetics of paclitaxel, which is also a substrate of CYP3A4, was not affected when given in combination with trastuzumab emtansine in breast cancer patients^32^. The greater than anticipated apparent oral clearance is probably an artifact of the small number of patients evaluated in the present study coupled with the high interpatient variability in the pharmacokinetics of the drug.

Our study adds to the growing body of clinical evidence suggesting a role for CDK 4/6 inhibitors in HER2+ MBC. Evidence to date has been strongest in HR+/HER2+ MBC. The phase 2 monarcHER trial was limited to HR+/HER2+ MBC^20^. The PATRICIA trial studying palbociclib 200 mg daily for 2 weeks and 1 week off plus trastuzumab had three cohorts, one for patients with HR-/HER2+ MBC and two for patients with HR+/HER2+ MBC (one with letrozole, one without)^22^. The progression-free survival rate at 6 months (PFS6) was lowest in the HR-/HER2+ cohort at 33.3% (5/15), followed by the HR+/HER2+ cohort without letrozole at 42.8% (12/28), with the highest PFS6 rate in the HR+/HER2+ cohort with letrozole at 46.4% (13/28)^22^. In the present study, 66.7% of patients had HR+/HER2+ MBC, suggesting the benefit of T-DM1 in combination with ribociclib can be seen regardless of hormone receptor status. Ongoing studies continue to explore the potential of CDK 4/6 inhibitors in HER2+ MBC. The randomized phase 3 PATINA study (NCT02947685) is assessing if the addition of palbociclib adds benefit to first-line trastuzumab, pertuzumab, and an aromatase inhibitor (after standard induction therapy with chemotherapy, trastuzumab, and pertuzumab) in patients with HR+/HER2+ MBC. Additional ongoing trials are evaluating the combination of palbociclib plus T-DM1 (NCT03530696) and tucatinib plus palbociclib and letrozole (NCT03054363).

With the increased use of CDK 4/6 inhibitors for HR+/HER2- MBC, there has also been a subsequent investigation into acquired resistance mechanisms following the use of these agents. Albeit a small sample size, the ctDNA samples from patients treated with the combination of ribociclib and T-DM1 in the present study are not consistent with established ER+ resistance mechanisms known to be acquired following the use of CDK 4/6 inhibitors^11,33^. This may be a function of the small sample size, though further investigation is warranted to understand if resistance to this regimen may be more strongly driven by T-DM1, compared to ribociclib. Understanding resistance mechanisms is critical with combination therapy to understand if either component may be useful in subsequent regimens. Continued use of an anti-HER2 backbone is a common approach in HER2+ MBC, but the potential for continued benefit from a CDK 4/6 inhibitor remains uncertain. The study results also highlight the role of a staggered dosing schedule with combination therapy with overlapping toxicities.

In conclusion, ribociclib in combination with T-DM1 in patients with HER2+ MBC demonstrated promising clinical activity and was generally well tolerated. The efficacy observed in this small study supports further investigation of CDK 4/6 inhibitors and anti-HER2 combinations.

## Methods

### Study design, and treatment plan

We conducted an open-label, phase Ib clinical trial designed to assess the safety, tolerability, and activity of ribociclib in combination with T-DM1 among women with metastatic or locally advanced HER2+ breast cancer. The study received institutional review board (IRB) approval from the Dana-Farber/Harvard Cancer Center Human Research Committee. The overall study (NCT02657343; registered January 16, 2016) included three separate cohorts: one cohort of ribociclib in combination with T-DM1 (cohort A), a second of ribociclib given in combination with trastuzumab (cohort B), and a third of ribociclib given in combination with trastuzumab and fulvestrant (cohort C). Here we report the results of the ribociclib plus T-DM1 cohort (cohort A).

T-DM1 was administered intravenously at the standard dose of 3.6 mg/kg on Day 1 of each 21-day cycle. A standard 3 + 3 dose-escalation design was used to evaluate various doses of ribociclib in combination with T-DM1. Ribociclib was administered orally starting at a dose of 300 mg daily on Days 8 through 21 of a 21-day cycle. The ribociclib dose was increased by 100 mg with each dose escalation, with the plan to reach but not exceed the current FDA-approved dose of single-agent ribociclib (600 mg). The dosing schedule of ribociclib was originally Days 5 through 18 of the 21-day cycle, however, was changed to Days 8 through 21 in Protocol Version #8 (November 28, 2016) due to thrombocytopenia in the setting of concurrent T-DM1. This change only impacted patients in the first dose level of ribociclib (300 mg). The protocol permitted concomitant bone-modifying agents and growth factors in accordance with the American Society of Clinical Oncology Guidelines^34^. Study treatment continued until radiographic or symptomatic disease progression, unacceptable toxicity, or withdrawal of informed consent.

The study was conducted in accordance with the International Conference on Harmonization Good Clinical Practice Guidelines (ICH GCP) and the Declaration of Helsinki, approved by the Dana Farber Cancer Institute’s institutional review board, and registered at ClinicalTrials.gov (NCT02657343). All patients provided written informed consent prior to the initiation of any study-related treatment or procedures.

### Patient population

Pre- and post-menopausal women aged 18 years or older with histologically confirmed unresectable, locally advanced, or metastatic HER2+ breast cancer and measurable or non-measurable disease according to Response Evaluation Criteria in Solid Tumor (RECIST) 1.1 were eligible^35^. HER2-positivity was defined by the 2013 American Society of Clinical Oncology-College of American Pathologists (ASCO-CAP) Guidelines^36^.

Patients must have had no more than 4 lines of prior systemic therapy in the metastatic setting, with at least one regimen containing trastuzumab and taxane in any setting. The protocol required patients to have an Eastern Cooperative Oncology Group performance score of ≤2; adequate bone marrow, liver, renal function; a left ventricular ejection fraction (LVEF) of at least 50% (determined by echocardiography (ECHO) or multiple-gated acquisition scan (MUGA)) at screening; adequate contraception if applicable; and a negative pregnancy test for women of childbearing potential. Patients with prior exposure to CDK4/6 inhibitors, QTcF >450 ms, or unstable brain metastases (untreated, symptomatic, or requiring steroids) were excluded. Prior treatment with T-DM1 was permissible unless T-DM1 had previously been discontinued due to disease progression or toxicity. For example, prior T-DM1 in the adjuvant setting was allowed.

### Safety and efficacy assessments

Adverse events were assessed at each patient visit and graded according to the National Cancer Institute Common Terminology Criteria for Adverse Events version 4.03. Cardiac toxicity was monitored every 4 cycles (12 weeks) utilizing the same modality used in screening (ECHO or MUGA) and by regular electrocardiographs (Cycle 1 Days 1, 8, 15, 21; Cycle 2 Days 8 and 15; and Day 1 of each subsequent cycle). Hematology and chemistry assessments were performed before Day 1 of each cycle. Ribociclib pharmacokinetic samples were collected on Cycle 1 Day 8, 9, 21, and Cycle 2 Day 1.

In the event of adverse events, treatment was interrupted or delayed and resumed only if protocol-defined criteria were met. Dose reductions, delays, and subsequent re-escalations were permitted as pre-specified by the protocol. Tumor assessments were performed at baseline and every 2 cycles (6 weeks) until disease progression or cycle 8, and then every 3 cycles (9 weeks) with the same imaging modality utilized in screening. Response and progression were evaluated based on RECIST 1.1^35^.

### Outcomes

The primary objective was to determine the maximum tolerated dose (MTD) and/or recommended phase 2 dose (RP2D) of ribociclib in combination with T-DM1. Secondary endpoints included evaluating the clinical activity (objective response rate and progression-free survival) in patients with HER2+ MBC treated with T-DM1 and ribociclib, as well as the pharmacokinetic profile of ribociclib in combination with T-DM1, and potential biomarkers of response to this regimen. Objective response rate (ORR) was defined as the portion of patients with complete response or partial response by RECIST 1.1 criteria. Progression-free survival (PFS) rate was defined as the time from study entry to first documented evidence of disease progression by RECIST 1.1 or death from any cause. All patients who received at least one dose of protocol therapy were evaluated for clinical benefit.

At the treating physician’s discretion, blood samples (10 ml using EDTA tubes) were collected for circulating tumor DNA (ctDNA) analysis prior to initiation of therapy, while on treatment, and at the time of progression. ctDNA analysis was performed using the Guardant360 assay, a commercially available, NGS-based assay that identifies potential tumor-related (somatic) genomic alterations via complete exon sequencing of critical exons within 73 cancer-related genes^37^.

### Pharmacokinetics

Blood samples (4 mL) for pharmacokinetic assessment were collected in plastic Vacutainer tubes with spray-dried K_2_EDTA (Becton, Dickinson and Co., Franklin Lakes, NJ) shortly before and at 0.5, 1.0, 2.0, 4.0, 6.0, 8.0, and 24.0 h after the first dose of ribociclib was given. Additional samples were collected according to this same schedule after the drug was taken for at least 8 days consecutive without interruption to define its steady-state pharmacokinetics. Blood samples were centrifuged within 30 min of collection to harvest the plasma which was promptly stored in cryovials maintained at −80 °C. The ribociclib concentration in plasma samples was determined using an analytical method based upon reversed-phase high-performance liquid chromatography with tandem mass spectrometric detection. The assay was validated and applied to the analysis of study samples according to the recommendations in the current U.S. Food and Drug Administration Guidance for Industry on Bioanalytical Method Validation (https://www.fda.gov/drugs/-guidance-compliance-regulatory-information/guidances-drugs). At the lowest concentration included in the calibration curves, which was 1.0 ng/mL, interday accuracy was 102.3% of the nominal concentration and the precision was 2.9%. Interday accuracy ranged from 96.1 to 104.3% and the precision ranged from 2.7 to 7.5% for all other calibration standards. Actual time points were calculated as the difference between the blood sample collection time and the time that the drug was taken. The plasma concentration-time data were analyzed by noncompartmental methods using model 200 for extravascular drug input in WinNonlin Professional version 5.0.1 (Pharsight Corp, Mountain View, CA). The area under the plasma concentration-time curve from the time of dosing to the time of the last sample collected prior to administration of the next dose (AUC24) was estimated using the linear-log trapezoidal method. Total oral clearance (CL/F) was calculated as the dose divided by the AUC24 for the dose given on days 19–21. Pharmacokinetic parameters are reported as the geometric mean (geometric CV%) of the values for individual patients at each dose level^38^. GraphPad Prism for Windows, version 8.4.3 (GraphPad Software, La Jolla, CA) was used for the statistical analysis and graphical presentation of the pharmacokinetic data.

### Statistical analysis

A standard 3 + 3 phase I design was employed where a minimum of three evaluable patients was accrued at the first dose level (300 mg ribociclib and 3.6 mg/kg IV T-DM1). If 1 out of the first 3 patients experienced dose-limiting toxicity (DLT), defined as any grade 3–4 non-hematological or grade 4 hematological toxicity at least possibly related to the treatment, occurring during the first cycle of treatment, three additional patients were accrued to the dose level. If no more than 1 patient of the 6 experienced a DLT, dose-escalation continued to the next dose level. If two or more patients at any given dose level experienced a DLT, dose escalation was halted and the maximum tolerated dose (MTD) was defined. We did not exceed the RP2D of the ribociclib (600 mg) single agent. The intention was to accrue at least 6 patients to treat at the MTD/RP2D of ribociclib and T-DM1 combination. A waterfall plot was generated to visualize patients’ response status and treatment duration. ORR was reported with a 90% confidence interval using the exact binomial method. PFS was defined as the time from study entry to the first documented evidence of disease progression by RECIST 1.1 or death from any cause, whichever occurred first. For this analysis, participants were considered to have progressed if they discontinued treatment with documented evidence of clinical deterioration due to breast cancer. Participants alive without disease progression were censored at the time of last disease evaluation. PFS was summarized using the Kaplan–Meier method. All participants were evaluated for toxicity from the time of their first treatment with any study agent. Toxicities were graded according to NCI CTCAE, Version 4.0. Maximum grade by type of toxicity was derived for each patient and tabulated. The datasets generated during and/or analyzed during the current study are available from the corresponding author on reasonable request.

### Reporting summary

Further information on research design is available in the Nature Research Reporting Summary linked to this article.

## Supplementary information

Supplementary Information

Reporting Summary

## Data Availability

The data generated and analyzed during this study are described in the following data record: 10.6084/m9.figshare.14822403^39^. All data are stored in the following files: Excel files “Fig. 1”, “Fig. 2”, “Fig. 3”, “Table 1”, “Table 2”; Word file: ‘Supplement TDM1 Ribo Manuscript_revision.docx’. The Excel files are housed on institutional file storage and are not publicly available in order to protect patient privacy as informed consent to share participant-level data was not obtained prior to or during data collection. However, the data can be made available upon request to the corresponding author. The Word file is shared publicly via the data record and is also available in PDF format as part of the supplementary materials of the article.
