# Supplementary material for: Phase 1b clinical trial of ado-trastuzumab emtansine and ribociclib for HER2-positive metastatic breast cancer
Source: NPJ Breast Cancer. 2021 Aug 4;7:103. doi: 10.1038/s41523-021-00311-y (PMC8339067; doi:10.1038/s41523-021-00311-y)
Supplement: Supplementary file 1 — Supplementary Information [file 41523_2021_311_MOESM1_ESM.pdf]

Supplementary Tables and Figures

Supplementary Table 1a. AEs related to treatment, dose level = 300mg

**Adverse event (AE) type**	**AE of any grade**	**Grade 1 AE**	**Grade 2 AE**	**Grade 3 AE**	**Grade 4 AE**
Anemia	3(100%)	1(33.33%)	1(33.33%)	1(33.33%)	0(0%)
Platelet count decreased	3(100%)	1(33.33%)	2(66.67%)	0(0%)	0(0%)
White blood cell decreased	3(100%)	1(33.33%)	1(33.33%)	1(33.33%)	0(0%)
Blood bilirubin increased	2(66.67%)	1(33.33%)	1(33.33%)	0(0%)	0(0%)
Diarrhea	2(66.67%)	2(66.67%)	0(0%)	0(0%)	0(0%)
Electrocardiogram QT corrected interval prolonged	2(66.67%)	2(66.67%)	0(0%)	0(0%)	0(0%)
Epistaxis	2(66.67%)	2(66.67%)	0(0%)	0(0%)	0(0%)
Nausea	2(66.67%)	2(66.67%)	0(0%)	0(0%)	0(0%)
Weight loss	2(66.67%)	2(66.67%)	0(0%)	0(0%)	0(0%)
Activated partial thromboplastin time prolonged	1(33.33%)	0(0%)	1(33.33%)	0(0%)	0(0%)
Alanine aminotransferase increased	1(33.33%)	1(33.33%)	0(0%)	0(0%)	0(0%)
Alkaline phosphatase increased	1(33.33%)	1(33.33%)	0(0%)	0(0%)	0(0%)
Aspartate aminotransferase increased	1(33.33%)	1(33.33%)	0(0%)	0(0%)	0(0%)
Bloating	1(33.33%)	1(33.33%)	0(0%)	0(0%)	0(0%)
Bruising	1(33.33%)	1(33.33%)	0(0%)	0(0%)	0(0%)
Cough	1(33.33%)	1(33.33%)	0(0%)	0(0%)	0(0%)
Cystitis noninfective	1(33.33%)	1(33.33%)	0(0%)	0(0%)	0(0%)
Dry eye	1(33.33%)	1(33.33%)	0(0%)	0(0%)	0(0%)
Dry mouth	1(33.33%)	1(33.33%)	0(0%)	0(0%)	0(0%)
Fatigue	1(33.33%)	0(0%)	1(33.33%)	0(0%)	0(0%)
Febrile neutropenia	1(33.33%)	0(0%)	0(0%)	1(33.33%)	0(0%)
Fever	1(33.33%)	0(0%)	1(33.33%)	0(0%)	0(0%)
Headache	1(33.33%)	1(33.33%)	0(0%)	0(0%)	0(0%)
Hypokalemia	1(33.33%)	1(33.33%)	0(0%)	0(0%)	0(0%)
Hypophosphatemia	1(33.33%)	0(0%)	1(33.33%)	0(0%)	0(0%)
Infusion related reaction	1(33.33%)	1(33.33%)	0(0%)	0(0%)	0(0%)
Lung infection	1(33.33%)	0(0%)	1(33.33%)	0(0%)	0(0%)
Lymphocyte count decreased	1(33.33%)	0(0%)	0(0%)	1(33.33%)	0(0%)
Mucositis oral	1(33.33%)	1(33.33%)	0(0%)	0(0%)	0(0%)
Myalgia	1(33.33%)	1(33.33%)	0(0%)	0(0%)	0(0%)
Neutrophil count decreased	1(33.33%)	0(0%)	1(33.33%)	0(0%)	0(0%)
Peripheral motor neuropathy	1(33.33%)	1(33.33%)	0(0%)	0(0%)	0(0%)
Urinary tract infection	1(33.33%)	0(0%)	0(0%)	1(33.33%)	0(0%)
Vomiting	1(33.33%)	1(33.33%)	0(0%)	0(0%)	0(0%)

Supplementary Table 1b. AEs related to treatment, dose level = 400mg

**Adverse event (AE) type**	**AE of any grade**	**Grade 1 AE**	**Grade 2 AE**	**Grade 3 AE**	**Grade 4 AE**
Fatigue	3(100%)	1(33.33%)	2(66.67%)	0(0%)	0(0%)
Neutrophil count decreased	3(100%)	0(0%)	2(66.67%)	0(0%)	1(33.33%)
Alanine aminotransferase increased	2(66.67%)	1(33.33%)	1(33.33%)	0(0%)	0(0%)
Anemia	2(66.67%)	1(33.33%)	1(33.33%)	0(0%)	0(0%)
Aspartate aminotransferase increased	2(66.67%)	1(33.33%)	1(33.33%)	0(0%)	0(0%)
Electrocardiogram QT corrected interval prolonged	2(66.67%)	2(66.67%)	0(0%)	0(0%)	0(0%)
Lymphocyte count decreased	2(66.67%)	1(33.33%)	1(33.33%)	0(0%)	0(0%)
Platelet count decreased	2(66.67%)	1(33.33%)	1(33.33%)	0(0%)	0(0%)
Rash maculo-papular	2(66.67%)	2(66.67%)	0(0%)	0(0%)	0(0%)
Alkaline phosphatase increased	1(33.33%)	1(33.33%)	0(0%)	0(0%)	0(0%)
Arthritis	1(33.33%)	1(33.33%)	0(0%)	0(0%)	0(0%)
Blood bilirubin increased	1(33.33%)	1(33.33%)	0(0%)	0(0%)	0(0%)
Constipation	1(33.33%)	1(33.33%)	0(0%)	0(0%)	0(0%)
Cough	1(33.33%)	1(33.33%)	0(0%)	0(0%)	0(0%)
Diarrhea	1(33.33%)	0(0%)	1(33.33%)	0(0%)	0(0%)
Gastroesophageal reflux disease	1(33.33%)	0(0%)	1(33.33%)	0(0%)	0(0%)
Gastrointestinal disorders - Other, specify	1(33.33%)	0(0%)	1(33.33%)	0(0%)	0(0%)
Hypophosphatemia	1(33.33%)	0(0%)	0(0%)	1(33.33%)	0(0%)
Nausea	1(33.33%)	1(33.33%)	0(0%)	0(0%)	0(0%)
Pain	1(33.33%)	1(33.33%)	0(0%)	0(0%)	0(0%)
Thrombotic thrombocytopenic purpura	1(33.33%)	0(0%)	1(33.33%)	0(0%)	0(0%)
Upper respiratory infection	1(33.33%)	0(0%)	1(33.33%)	0(0%)	0(0%)
White blood cell decreased	1(33.33%)	1(33.33%)	0(0%)	0(0%)	0(0%)

Supplemental Table 1c. AEs related to treatment dose level = 500mg

**Adverse event (AE) type**	**AE of any grade**	**Grade 1 AE**	**Grade 2 AE**	**Grade 3 AE**	**Grade 4 AE**
Neutrophil count decreased	3(100%)	0(0%)	1(33.33%)	2(66.67%)	0(0%)
Platelet count decreased	3(100%)	2(66.67%)	0(0%)	1(33.33%)	0(0%)
Anemia	2(66.67%)	2(66.67%)	0(0%)	0(0%)	0(0%)
Aspartate aminotransferase increased	2(66.67%)	1(33.33%)	1(33.33%)	0(0%)	0(0%)
White blood cell decreased	2(66.67%)	0(0%)	1(33.33%)	1(33.33%)	0(0%)
Alanine aminotransferase increased	1(33.33%)	1(33.33%)	0(0%)	0(0%)	0(0%)
Alopecia	1(33.33%)	1(33.33%)	0(0%)	0(0%)	0(0%)
Bruising	1(33.33%)	1(33.33%)	0(0%)	0(0%)	0(0%)
Cough	1(33.33%)	1(33.33%)	0(0%)	0(0%)	0(0%)
Dry mouth	1(33.33%)	0(0%)	1(33.33%)	0(0%)	0(0%)
Fatigue	1(33.33%)	1(33.33%)	0(0%)	0(0%)	0(0%)
Hyperhidrosis	1(33.33%)	1(33.33%)	0(0%)	0(0%)	0(0%)
Mucositis oral	1(33.33%)	1(33.33%)	0(0%)	0(0%)	0(0%)
Nausea	1(33.33%)	1(33.33%)	0(0%)	0(0%)	0(0%)

Supplementary Table 1d. AE related to treatment, dose level = 600mg

**Adverse event (AE) type**	**AE of any grade**	**Grade 1 AE**	**Grade 2 AE**	**Grade 3 AE**	**Grade 4 AE**
Anemia	3(100%)	0(0%)	1(33.33%)	2(66.67%)	0(0%)
Platelet count decreased	3(100%)	1(33.33%)	1(33.33%)	1(33.33%)	0(0%)
Creatinine increased	2(66.67%)	0(0%)	2(66.67%)	0(0%)	0(0%)
Fatigue	2(66.67%)	2(66.67%)	0(0%)	0(0%)	0(0%)
Lymphocyte count decreased	2(66.67%)	1(33.33%)	0(0%)	1(33.33%)	0(0%)
Neutrophil count decreased	2(66.67%)	0(0%)	0(0%)	2(66.67%)	0(0%)
White blood cell decreased	2(66.67%)	0(0%)	0(0%)	2(66.67%)	0(0%)
Alanine aminotransferase increased	1(33.33%)	0(0%)	0(0%)	1(33.33%)	0(0%)
Aspartate aminotransferase increased	1(33.33%)	0(0%)	0(0%)	1(33.33%)	0(0%)
Electrocardiogram QT corrected interval prolonged	1(33.33%)	0(0%)	1(33.33%)	0(0%)	0(0%)
Epistaxis	1(33.33%)	1(33.33%)	0(0%)	0(0%)	0(0%)
Fever	1(33.33%)	0(0%)	1(33.33%)	0(0%)	0(0%)
Mucositis oral	1(33.33%)	1(33.33%)	0(0%)	0(0%)	0(0%)
Nausea	1(33.33%)	0(0%)	1(33.33%)	0(0%)	0(0%)
Oral hemorrhage	1(33.33%)	1(33.33%)	0(0%)	0(0%)	0(0%)
Renal and urinary disorders - Other, specify	1(33.33%)	0(0%)	0(0%)	1(33.33%)	0(0%)
Salivary duct inflammation	1(33.33%)	1(33.33%)	0(0%)	0(0%)	0(0%)
Vomiting	1(33.33%)	0(0%)	1(33.33%)	0(0%)	0(0%)

Supplementary Table 2. Ribociclib pharmacokinetic parameters.

Ribociclib pharmacokinetic parameters[a]										
Dose (mg)	Dose no.	No. of patients	C_{min} (ng/mL)		C_{max} (ng/mL)		AUC_{24} (ng•h/mL)		CL/F (L/h)	
300	1	3	<LLQ		504.8	(20.4)	5,327	(7.0)		
300	11-12	3	141.7	(49.0)	608.3	(21.6)	7,767	(16.4)	38.6	(16.4)
400	1	3	<LLQ		734.8	(53.4)	7,979	(44.6)		
400	13	1	125.6		870.7		8,431		47.4	
500	1	3	<LLQ		577.2	(55.9)	4,831	(33.8)		
500	12	2	178.7	(39.2)	702.8	(3.8)	8,958	(14.6)	55.8	(14.6)
600	1	3	<LLQ		1,272	(46.9)	11,814	(55.7)		
600	13	1	493.9		1,743		21,975		27.3	

[a] Data are reported as the geometric mean (CV%).

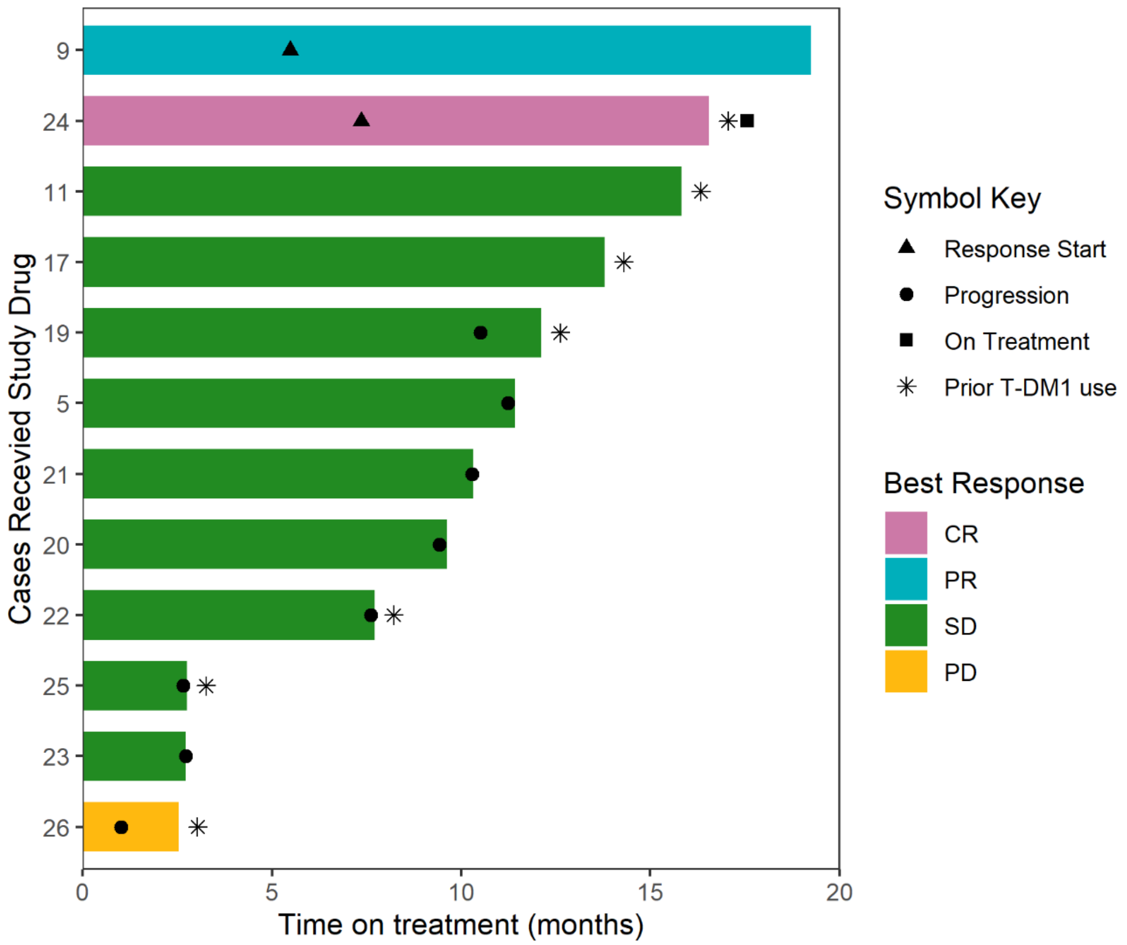

Supplementary Figure 1. Swimmer plot, stratified by prior T-DM1 use.

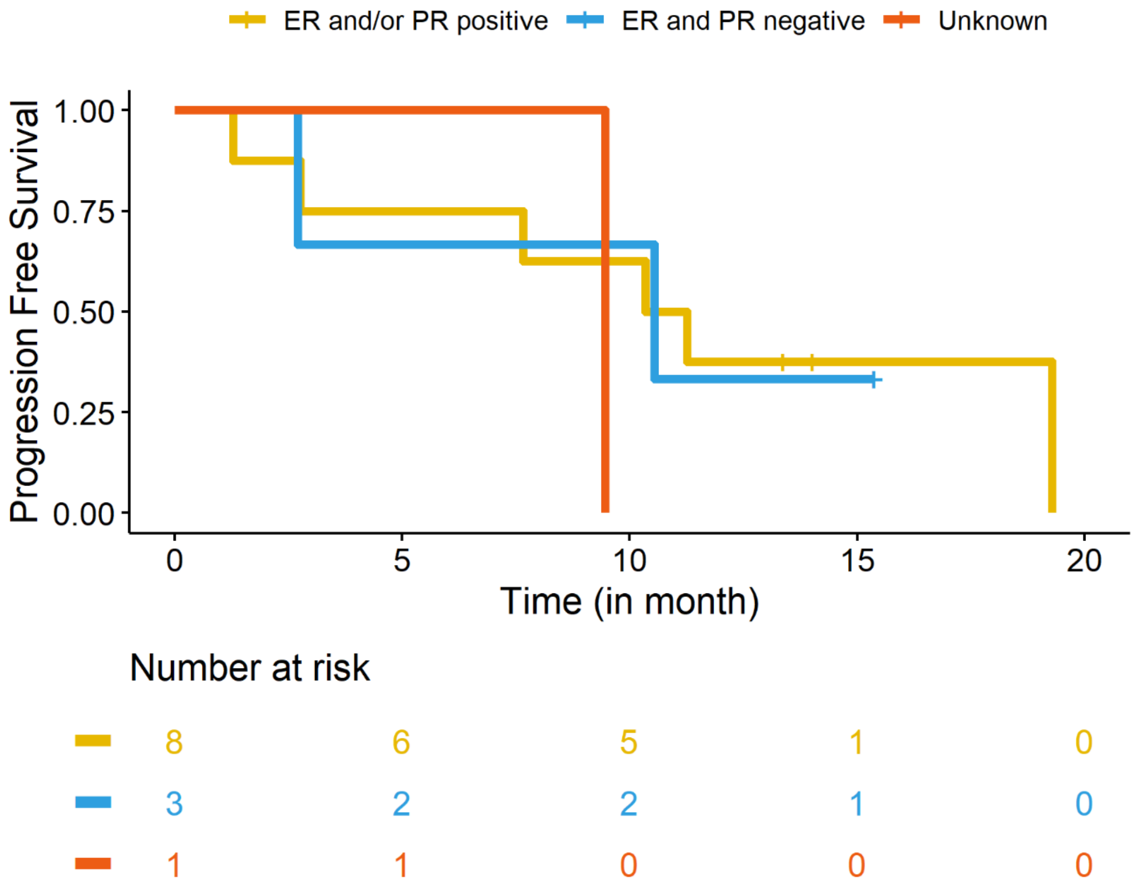

Supplementary Figure 2. Median progression free survival (PFS) summarized in a Kaplan-Meier plot, stratified by hormone receptor (HR) status. The median PFS for patients with and without HR positivity was 10.8 months (95% CI [1.3-19.3]) and 10.5 months (95% CI [2.7-not reached]), respectively.

Subject ID	005			009			011			017			019			020			022			023			025		
**Diagnosis (De Novo vs. Recurrence)**	Recurrence			Recurrence			De Novo			Recurrence			De Novo			De Novo			Recurrence			Recurrence			Recurrence		
**Prior Lines Metastatic Setting (#)**	1			0			2			3			3			1			2			2			2		
**Time on Study (days)**	342			643			511			420			287			308			235			84			84		
**Timepoint**	Pre -Tx	On Tx	Post -Tx	Pre -Tx	On Tx	Post -Tx	Pre -Tx	On Tx	Post-Tx	Pre -Tx	On Tx	Post -Tx	Pre-Tx	On Tx	Post-Tx	Pre-Tx	On Tx	Post-Tx	Pre -Tx	On Tx	Post -Tx	Pre -Tx	On Tx	Post -Tx	Pre -Tx	On Tx	Post -Tx
		C5	N/A		C4			C3	No mutations detected		C3	N/A		N/A			C2			N/A			C3	N/A		N/A	N/A
**Alterations**																											
APC		0.1															0.1				1.2						
AR																									0.1		
AR Amp																+											
ARID1A		0.1																							1.6		
ATM																0.2	0.3	0.3									
BRAF																1.5	1.6	1.1									
BRCA2										0.2						0.5											
CCND1 Amp																++											
EGFR Amp																			+++		+						
ERBB2	2.7	0.5		0.1																			0.2				
ERBB2 Amp				+												+++							++		++		
FGFR1 Amp																+++											
FGFR2 Amp																		0.1									
GNAS																						0.2	0.2				
KIT																									1.3		
MYC Amp																									+		
NRAS				0.4		0.4																					
PIK3CA																0.7			3.6		3.9				1.7		
RB1 Splice Site																		0.3									
TP53							0.2	0.1					R175G: 0.1		P82fs: 0.3 A88fs:0.2	P316fs:19.9	N131S: 0.1 P316fs: ND	N581S: 1.1 N131S: 0.5 R273H: 0.3				0.3	1.7		4.3		

Supplementary Figure 3: Circulating tumor (ct) DNA analysis

*N/A Indicates sample was not collected. Three patients had no samples collected and were therefore excluded from this figure.
