# Supplementary material for: Phase 1b clinical trial of ado-trastuzumab emtansine and ribociclib for HER2-positive metastatic breast cancer
Source: NPJ Breast Cancer. 2021 Aug 4;7:103. doi: 10.1038/s41523-021-00311-y (PMC8339067; doi:10.1038/s41523-021-00311-y)
Supplement: Supplementary file 2 — Reporting Summary [file 41523_2021_311_MOESM2_ESM.pdf]

nature research

Corresponding author(s): *Double-blind peer review submissions: write DBPR and your manuscript number here instead of author names.*

Last updated by author(s): *YYYY-MM-DD*

Reporting Summary

Nature Research wishes to improve the reproducibility of the work that we publish. This form provides structure for consistency and transparency in reporting. For further information on Nature Research policies, see our Editorial Policies and the Editorial Policy Checklist.

Statistics

For all statistical analyses, confirm that the following items are present in the figure legend, table legend, main text, or Methods section.

n/a	Confirmed	
☐	☒	The exact sample size (*n*) for each experimental group/condition, given as a discrete number and unit of measurement
☐	☒	A statement on whether measurements were taken from distinct samples or whether the same sample was measured repeatedly
☐	☒	The statistical test(s) used AND whether they are one- or two-sided<br>*Only common tests should be described solely by name; describe more complex techniques in the Methods section.*
☐	☒	A description of all covariates tested
☐	☒	A description of any assumptions or corrections, such as tests of normality and adjustment for multiple comparisons
☐	☒	A full description of the statistical parameters including central tendency (e.g. means) or other basic estimates (e.g. regression coefficient) AND variation (e.g. standard deviation) or associated estimates of uncertainty (e.g. confidence intervals)
☒	☐	For null hypothesis testing, the test statistic (e.g. *F*, *t*, *r*) with confidence intervals, effect sizes, degrees of freedom and *P* value noted<br>*Give P values as exact values whenever suitable.*
☒	☐	For Bayesian analysis, information on the choice of priors and Markov chain Monte Carlo settings
☒	☐	For hierarchical and complex designs, identification of the appropriate level for tests and full reporting of outcomes
☒	☐	Estimates of effect sizes (e.g. Cohen's *d*, Pearson's *r*), indicating how they were calculated

Our web collection on statistics for biologists contains articles on many of the points above.

Software and code

Policy information about availability of computer code

Data collection

We conducted an open-label, phase Ib clinical trial designed to assess the safety, tolerability, and activity of ribociclib in combination with T-DM1 among women with metastatic or locally advanced HER2+ breast cancer. The overall study (NCT02657343; registered Jan 16th 2016) included 3 separate cohorts: one cohort of ribociclib in combination with T-DM1 (cohort A), a second of ribociclib given in combination with trastuzumab (cohort B), and a third of ribociclib given in combination with trastuzumab and fulvestrant (cohort C). Here we report the results of the ribociclib plus T-DM1 cohort (cohort A). Pre- and post-menopausal women aged 18 years or older with histologically confirmed unresectable, locally advanced, or metastatic HER2+ breast cancer and measurable or non-measurable disease according to Response Evaluation Criteria in Solid Tumor (RECIST) 1.1 were eligible and enrolled in the study. The study was conducted in accordance with the International Conference on Harmonization Good Clinical Practice Guidelines (ICH GCP) and the Declaration of Helsinki, approved by the Dana Farber Cancer Institute's institutional review board, and registered at ClinicalTrials.gov (NCT02657343). All patients provided written informed consent prior to the initiation of any study-related treatment or procedures.

Data analysis

A standard 3+3 phase I design was employed where a minimum of 3 evaluable patients were accrued at the first dose level (300 mg ribociclib and 3.6 mg/kg IV T-DM1). If 1 out of the first 3 patients experienced a dose-limiting toxicity (DLT), defined as any grade 3–4 non-hematological or grade 4 hematological toxicity at least possibly related to the treatment, occurring during the first cycle of treatment, 3 additional patients were accrued to the dose level. If no more than 1 patient of the 6 experienced a DLT, dose escalation continued to the next dose level. If two or more patients at any given dose level experienced a DLT, dose escalation was halted and the maximum tolerated dose (MTD) was defined. We did not exceed the RP2D of ribociclib (600 mg) single agent. The intention was to accrue at least 6 patients to treat at the MTD/RP2D of ribociclib & T-DM1 combination. A waterfall plot was generated to visualize patients' response status and treatment duration. ORR was reported with 90% confidence interval using the exact binomial method. PFS was defined as the time from study entry to the first documented evidence of disease progression by RECIST 1.1 or death from any cause, whichever occurred first. For this analysis, participants were considered to have progressed if they discontinued treatment with documented evidence of clinical deterioration due to breast cancer. Participants alive without disease progression were censored at the time of last disease evaluation. PFS was summarized using the Kaplan-Meier method. All participants were evaluated for toxicity from the time of their first treatment with any study agent. Toxicities were graded

according to NCI CTCAE, Version 4.0. Maximum grade by type of toxicity was derived for each patient and tabulated.

For manuscripts utilizing custom algorithms or software that are central to the research but not yet described in published literature, software must be made available to editors and reviewers. We strongly encourage code deposition in a community repository (e.g. GitHub). See the Nature Research guidelines for submitting code & software for further information.

Data

Policy information about availability of data

All manuscripts must include a data availability statement. This statement should provide the following information, where applicable:

- Accession codes, unique identifiers, or web links for publicly available datasets
- A list of figures that have associated raw data
- A description of any restrictions on data availability

The datasets generated during and/or analyzed during the current study are available from the corresponding author on reasonable request.

Field-specific reporting

Please select the one below that is the best fit for your research. If you are not sure, read the appropriate sections before making your selection.

[x] Life sciences [ ] Behavioural & social sciences [ ] Ecological, evolutionary & environmental sciences

For a reference copy of the document with all sections, see nature.com/documents/nr-reporting-summary-flat.pdf

Life sciences study design

All studies must disclose on these points even when the disclosure is negative.

Sample size	A standard 3+3 phase I design was employed where a minimum of 3 evaluable patients were accrued at the first dose level (300 mg ribociclib and 3.6 mg/kg IV T-DM1). If 1 out of the first 3 patients experienced a dose-limiting toxicity (DLT), defined as any grade 3–4 non-hematological or grade 4 hematological toxicity at least possibly related to the treatment, occurring during the first cycle of treatment, 3 additional patients were accrued to the dose level. If no more than 1 patient of the 6 experienced a DLT, dose escalation continued to the next dose level. If two or more patients at any given dose level experienced a DLT, dose escalation was halted and the maximum tolerated dose (MTD) was defined. We did not exceed the RP2D of ribociclib (600 mg) single agent. The intention was to accrue at least 6 patients to treat at the MTD/RP2D of ribociclib & T-DM1 combination
Data exclusions	No specific exclusion for analysis. All eligible patients were included in intention to treat analysis.
Replication	Not applicable
Randomization	Not applicable
Blinding	Not applicable

Reporting for specific materials, systems and methods

We require information from authors about some types of materials, experimental systems and methods used in many studies. Here, indicate whether each material, system or method listed is relevant to your study. If you are not sure if a list item applies to your research, read the appropriate section before selecting a response.

Materials & experimental systems

n/a	Involved in the study	
[x]	[ ]	Antibodies
[x]	[ ]	Eukaryotic cell lines
[x]	[ ]	Palaeontology and archaeology
[x]	[ ]	Animals and other organisms
[x]	[ ]	Human research participants
[ ]	[x]	Clinical data
[x]	[ ]	Dual use research of concern

Methods

n/a	Involved in the study	
[x]	[ ]	ChIP-seq
[x]	[ ]	Flow cytometry
[x]	[ ]	MRI-based neuroimaging

Clinical data

Policy information about clinical studies

All manuscripts should comply with the ICMJE guidelines for publication of clinical research and a completed CONSORT checklist must be included with all submissions.

Clinical trial registration	NCT02657343

Study protocol	The protocol and datasets generated during and/or analyzed during the current study are available from the corresponding author on reasonable request.
Data collection	Between March 9, 2016 and May 25, 2019, a total of 12 patients were enrolled.
Outcomes	ORR was reported with 90% confidence interval using the exact binomial method. PFS was defined as the time from study entry to the first documented evidence of disease progression by RECIST 1.1 or death from any cause, whichever occurred first. For this analysis, participants were considered to have progressed if they discontinued treatment with documented evidence of clinical deterioration due to breast cancer. Participants alive without disease progression were censored at the time of last disease evaluation. PFS was summarized using the Kaplan-Meier method. All participants were evaluated for toxicity from the time of their first treatment with any study agent. Toxicities were graded according to NCI CTCAE, Version 4.0.
